# A Case of Perforation of the Pericardium by Echinococci

**Published:** 1859-01

**Authors:** 


*A Case of Perforation of the Pericardium by Echinococci.* By C. A. Wunderlich. (Archiv für Physiol. Heilkunde, Jahrgang, 1858. Zweites Heft.)

A laboring man, aged twenty-two, had always enjoyed good health, with the exception of an attack of peritonitis following a blow on the abdomen when sixteen years old. In June, 1857, he observed that his trousers were getting too tight for him across the belly, without other unpleasant symptoms. A week later he was attacked with cholic, diarrhœa, headache, vertigo, and thirst; rigors followed, and he was brought to the hospital. The heart and lungs appeared healthy, but the diaphragm was pushed up to the fifth rib; there was high fever. The abdomen was tender, and the hypogastric region covered with a venous plexus; one prominence was observed in the epigastrium, and another in the cæcal region, due to movable tumors. The former yielded a hollow percussion-sound, the latter one resembling the vibration characteristic of hydatids; the tumors were not more tender than the rest of the abdomen. The total evidence spoke rather in favor of the presence of cancer than of hydatid cysts. The tumors grew rapidly, the tenderness increased, the fever persisted, and icterus supervened, with severe epistaxis and hæmatemesis. Some improvement took place after the middle of the ensuing July, and the patient in August began to leave his bed; the idea of the cancerous nature of the tumors therefore was abandoned. On the 22d September there was a temporary relapse, and on the 28th September severe pain in the abdomen, and dyspnœa, with great tenderness of the upper tumor. The patient recovered again somewhat, but the symptoms fluctuated more or less till the 18th October, when there was a sudden fall of temperature of the body, contracted features, cold sweats, small, slow pulse, quick breathing, increasing collapse, and death on the 20th October. We only note the prominent points observed in the autopsy twenty-nine hours later. In a pulmonary artery of third order, of the inferior right lobe, there was an echinococcous cyst of the size of a pigeon's egg; the remains of echinococci were found in the branches given off from this artery. Pleura healthy; pericardium distended up to the second rib, containing four ounces of a purulent fluid. The parietal layer was thickened and covered with yellowish-red villi; the visceral layer was 1½ line thick; the heart reduced in size, its tissue pale and very friable. At the base of the pericardium there was a perforation with thin, smooth edges, which was covered by the heart, and which passed through the diaphragm, establishing a communication between the cavity of the pericardium and the epigastric tumor; the perforation was blocked up by a small echinococcous cyst which had got wedged into it. The left lobe of the liver was almost entirely replaced by a large hydatid tumor of the size of a child's head, containing numerous subdivisions with echinococci; otherwise, there was no marked derangement in the liver. The upper third of the spleen was occupied by a hydatid tumor of the size of a fist; in the retro-peritoneal space between the diaphragm and the stomach were three similar tumors of the size of apples: six were also found, from the size of a walnut to that of an apple, in the omentum. Between the psoas and the posterior surface of the cæcum was one of the size of a fist; a cylindrical one, three inches long and one broad, lay across the hypogastrium; above fifty were scattered over the mesentery, and two lay under the serous investment of the vermiform process. The intestinal mucous membrane was normal, there was no ascites, and nothing marked about the kidneys.—*British and Foreign Medico-Chirurgical Review.*

